# Supplementary material for: Cell non-autonomous control of autophagy and metabolism by glial cells
Source: iScience. 2024 Feb 28;27(4):109354. doi: 10.1016/j.isci.2024.109354 (PMC10946330; doi:10.1016/j.isci.2024.109354)
Supplement: Document S1. Figures S1–S4 and Table S1 [file mmc1.pdf]

iScience, Volume *27*

Supplemental information

Cell non-autonomous control of autophagy and metabolism by glial cells

Melissa G. Metcalf, Samira Monshietehadi, Arushi Sahay, Jenni Durieux, Ashley E. Frakes, Martina Velichkovska, Cesar Mena, Amelia Farinas, Melissa Sanchez, and Andrew Dillin

SUPPLEMENTAL FIGURE LEGENDS

Figure S1 Glial XBP-1s animals have reduced lipid content, not due to differences in autofluorescence or food intake, related to Figure 1.

(A) Quantification of fixed, unstained wild-type and glial XBP-1s animals using a COPAS BioSorter to determine whole worm autofluorescence quantification. Animals were grown to day 2 of adulthood on OP50 bacteria. Intensity of green values was compared to mean of fixed, unstained wild-type animals to determine fold change. Box plot shows median, whiskers are minimum to maximum values. N > 100 animals per condition. Statistics by Mann-Whitney test, p < 0.0001 (****).

(B) Quantification of wild-type, glial XBP-1s, *eat-2*(*e1372*), and *daf-7*(*ad1116*) animals at day 2 of adulthood stained with BODIPY 493/503 using a COPAS BioSorter for whole worm BODIPY 493/503 dye quantification. BODIPY 493/503 staining intensity was compared to mean of wild-type animals to determine fold change. Box plot shows median, whiskers are minimum to maximum values. N > 100 animals per condition. Statistics by One-way ANOVA with Sidák's multiple comparison test, p < 0.0001 (****), p < 0.5 (*).

(C) Quantification of wild-type and glial XBP-1s using a COPAS BioSorter to determine whole worm autofluorescence quantification. Animals were grown to day 2 of adulthood on OP50 bacteria. Intensity of autofluorescence was compared to mean of wild-type animals to determine fold change. Box plot shows median, whiskers are minimum to maximum values. N > 100 animals per condition. Statistics by Mann-Whitney test, p > 0.05 (ns = not significant).

(D) Pharyngeal pumping per minute of wild-type, glial XBP-1s (integrated strain #1), and glial XBP-1s (integrated strain #2) animals at the young adult stage. The graph shows representative data of two experiments, n = 10 animals per condition. Statistics by Kruskal-Wallis with Dunn's multiple comparison test, p > 0.05 (ns).

(E) Representative fluorescent micrograph of wild-type and glial XBP-1s animals transgenic for VIT-2::GFP at day 1 of adulthood grown on OP50 *E. coli*. White boxes indicate eggs within the adult animal. Scale bar, 250 μM.

(F) Quantification of rough ER cisternae space circularity in wild-type and glial XBP-1s animals from Figure 1J. A circularity value of 1.0 indicates a perfect circle. As the value approaches 0.0, it indicates an increasingly elongated polygon. Plots are of measurements of rough ER from n > 15 samples over 3 independent replicates. Statistics by Mann-Whitney test, p < 0.0001 (****).

Figure S2 Glial XBP-1s animals have upregulated levels of fluorescently tagged DAF-16 protein, related to Figure 2.

(A) Representative fluorescent micrograph of wild-type and glial XBP-1s animals transgenic for DAF-16::GFP. Animals were imaged at day 1 of adulthood. Images are representative of three independent replicates. Scale bar, 250 μM.

(B) Quantification of animals in (A) using a COPAS BioSorter for whole worm fluorescence quantification. Fluorescence intensity was compared to mean of wild-type animals to determine fold change. Box plot shows median, whiskers are minimum to maximum values. Plot is representative data of three independent replicates, N > 500 animals per condition. Statistics by Kruskal-Wallis with Dunn's multiple comparison test, $p < 0.0001$ (****).

Figure S3 HLH-30 nuclear localization is not impacted by *unc-31*, *unc-13*, or *xbp-1*, related to Figure 3.

(A) Representative fluorescent micrograph of wild-type and *unc-31(e928)* mutant animals transgenic for HLH-30::GFP treated with a heat shock exposure at 35ºC for 3 hours. Scale bar, 250 μM. Image is representative of 2 independent replicates. All animals imaged showed nuclear localization, N > 20 per condition.

(B) Nuclear translocation of HLH-30::GFP in intestinal cells of day 2 adult wild-type and glial XBP-1s animals fed OP50 *E. coli* with and without the loss-of-function *unc-13* mutant, *unc-13(s69)*, which disrupts both isoforms of *unc-13.* N > 36 animals per condition from three independent replicates. Statistics done by Chi-squared test for independence with adjusted residuals and Bonferroni correction, statistics shown in Table S4.

(C) qRT-PCR analysis of HLH-30 autophagy and lysosomal target genes from whole animal samples of wild-type animals treated with DMSO or tunicamycin. Bar graph shows mean transcript levels normalized to expression in wild-type animals treated with DMSO, from three independent biological and four technical replicates. Error bars represent SEM. Statistics by Two-way ANOVA with Sidák's multiple comparison test, $p > 0.05$ (ns). All comparisons were found to be non-significant.

(D) Nuclear translocation of HLH-30::GFP in intestinal cells of day 2 adult wild-type and glial XBP-1s animals fed OP50 *E. coli* with and without the loss-of-function *xbp-1* mutant, *xbp-1(tm2492).* N > 33 animals per condition from three independent replicates. Statistics done by Chi-squared test for independence with adjusted residuals and Bonferroni correction, statistics shown in Table S4.

(E) Nuclear translocation of HLH-30::GFP in intestinal cells of day 2 adult wild-type and extrachromosomal array glial HLH-30 (*hlh-17*p::*hlh-30*) fed OP50 *E. coli*. N > 30 animals per strain from two independent replicates.

(F) Survival of wild-type and glial HLH-30 (*hlh-17*p::*hlh-30*, extra chromosomal arrays) animals on control RNAi at 20ºC. Graph was plotted as Kaplan-Meier survival curves and p values were calculated by Mantel-Cox log-rank test. See Table S1 for lifespan statistics.

(G) Comparison of log2(fold change) of the 86 upregulated DEGs in glial XBP-1s animals compared to wild-type animals (top) compared to the log2(fold change) of these genes in glial XBP-1s animals with a loss-of-function *hlh-30* mutation, *hlh-30(tm1978),* compared to expression in *hlh-30(tm1978)* mutants alone (bottom). log2(fold change) is color-coded via a heatmap from warm (up-regulated) to cool (down-regulated) colors. Green dots above the heat map represent HLH-30 target genes. Statistics shown in Table S5.

Figure S4 Impact of *hlh-30, egl-3, bec-1,* and *unc-31* on glial XBP-1s phenotypes, related to Figure 4 and Figure 5.

(A) Quantification of mCherry puncta co-localized with GFP (autophagosomes (AP)) intestine of wild-type and glial XBP-1s transgenic animals at day 2 of adulthood. Data are from three independent experiments, each with ≥ 5 animals. Statistics by Kruskal-Wallis with Dunn's multiple comparison test, $p < 0.01$ (**), $p < 0.5$ (*), $p > 0.5$ (ns).

(B) Quantification of puncta containing mCherry alone (autolysosome (AL)) (in the intestine of wild-type and glial XBP-1s transgenic animals at day 2 of adulthood. Data are from three independent experiments, each with ≥ 5 animals. Statistics by Kruskal-Wallis with Dunn's multiple comparison test, $p < 0.0001$ (****), $p > 0.5$ (ns).

(C) qRT-PCR analysis of HLH-30 autophagy and lysosomal target genes in whole animal samples from wild-type and glial XBP-1s animals. Bar graph represents mean transcript levels normalized to wild-type control, from three independent biological and four technical replicates. Error bars represent SEM. Statistics by Two-way ANOVA with Sidák's multiple comparison test, $p < 0.01$ (**), $p < 0.0001$ (****).

(D) Fluorescence intensity of intestinal $polyQ_{44}$ aggregates from wild-type and glial XBP-1s animals grown on control or *hlh-30* RNAi quantified from Figure 4F using ImageJ and expressed as fluorescence intensity relative to the average intensity of wild-type animals expressing $polyQ_{44}$. Box plot shows median, whiskers are minimum to maximum values. Statistics by Kruskal-Wallis with Dunn's multiple comparison test $p < 0.001$ (***), $p < 0.0001$ (****). N = 50 animals per condition.

(E) qRT-PCR analysis of HLH-30 autophagy and lysosomal target genes in whole animal samples from wild-type and *unc-31(e928)* animals. Bar graph represents mean transcript levels normalized to wild-type control, from three independent biological and four technical replicates. Error bars represent SEM. Statistics by Two-way ANOVA with with Sidák's multiple comparison test, $p < 0.01$ (**), $p <0.001$ (***), $p <0.0001$ (****).

(F) Representative micrographs from intestine of wild-type and glial XBP-1s animals transgenic for the ER marker *vha-6p*::ERss::mRuby::HDEL with or without *egl-3(ok979)* or *unc-31(e928)* mutations at day 3 of adulthood. Animals were grown on OP50 *E. coli*. ER puncta is denoted by white arrowheads.

(G) Quantification of ER puncta from Figure S4F in wild-type and glial XBP-1s animals transgenic for the ER marker *vha-6p*::ERss::mRuby::HDEL with or without *egl-3(ok979)* or *unc-31(e928)* mutations at day 3 of adulthood. Plots are of measurements of mRuby::HDEL puncta from $n > 15$ samples over 3 independent experiments. Statistics by Kruskal-Wallis with Dunn's multiple comparison test, $p < 0.0001$ (****), $p < 0.01$ (**), $p < 0.05$ (*), $p > 0.5$ (ns).

(H) Quantification of rough ER cisternae space circularity from Figure 5G in wild-type and glial XBP-1s animals fed control or *bec-1* RNAi. A circularity value of 1.0 indicates a perfect circle. As the value approaches 0.0, it indicates an increasingly elongated polygon. Plots are of measurements of rough ER from $n > 15$ samples over 3 independent experiments. Statistics by Kruskal-Wallis with Dunn's multiple comparison test, $p < 0.0001$ (****), $p > 0.5$ (ns).

Figure S1

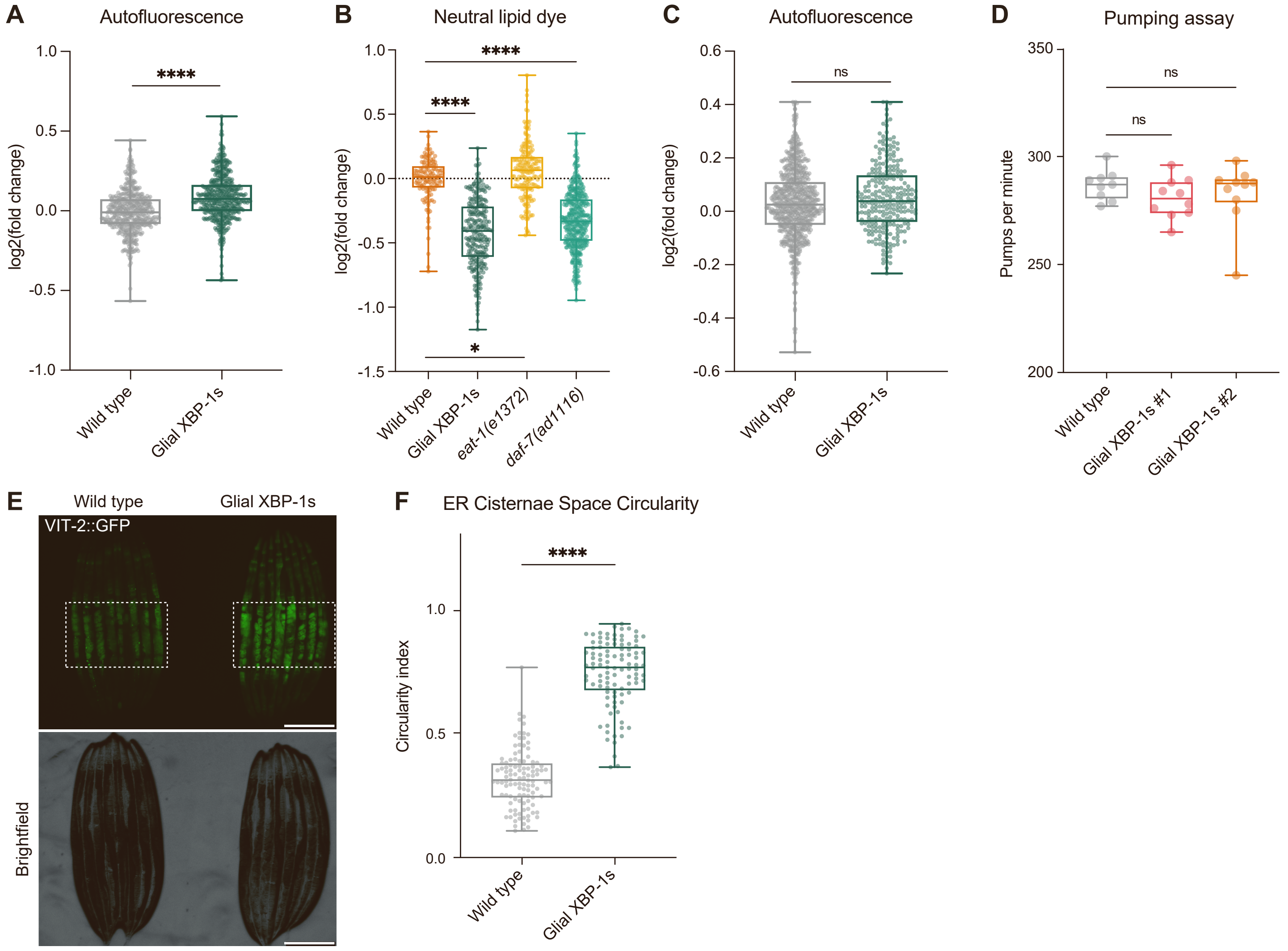

A
Autofluorescence
log2(fold change)
1.0
0.5
0.0
-0.5
-1.0
****
Wild type
Glial XBP-1s
B
Neutral lipid dye
log2(fold change)
1.0
0.5
0.0
-0.5
-1.0
-1.5
****
****
*
Wild type
Glial XBP-1s
eat-1(e1372)
daf-7(ad1116)
C
Autofluorescence
log2(fold change)
0.6
0.4
0.2
0.0
-0.2
-0.4
-0.6
ns
Wild type
Glial XBP-1s
D
Pumping assay
Pumps per minute
350
300
250
200
ns
ns
Wild type
Glial XBP-1s #1
Glial XBP-1s #2
E
Wild type
Glial XBP-1s
VIT-2::GFP
Brightfield
F
ER Cisternae Space Circularity
Circularity index
1.0
0.5
0.0
****
Wild type
Glial XBP-1s

Figure S2

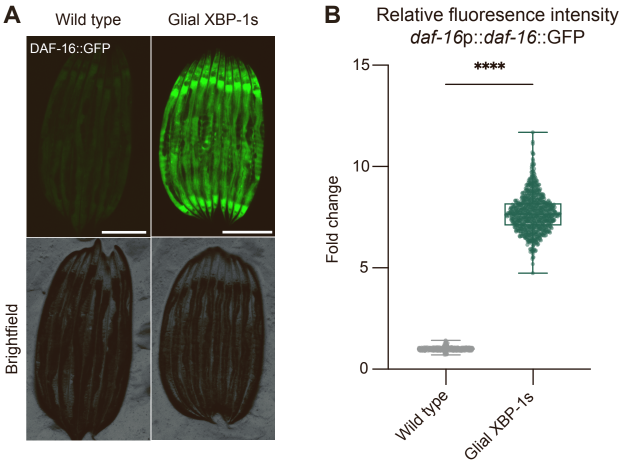
A
Wild type
Glial XBP-1s
DAF-16::GFP
Brightfield
B
Relative fluoresence intensity
daf-16p::daf-16::GFP
****
15
10
5
0
Fold change
Wild type
Glial XBP-1s

Figure S3

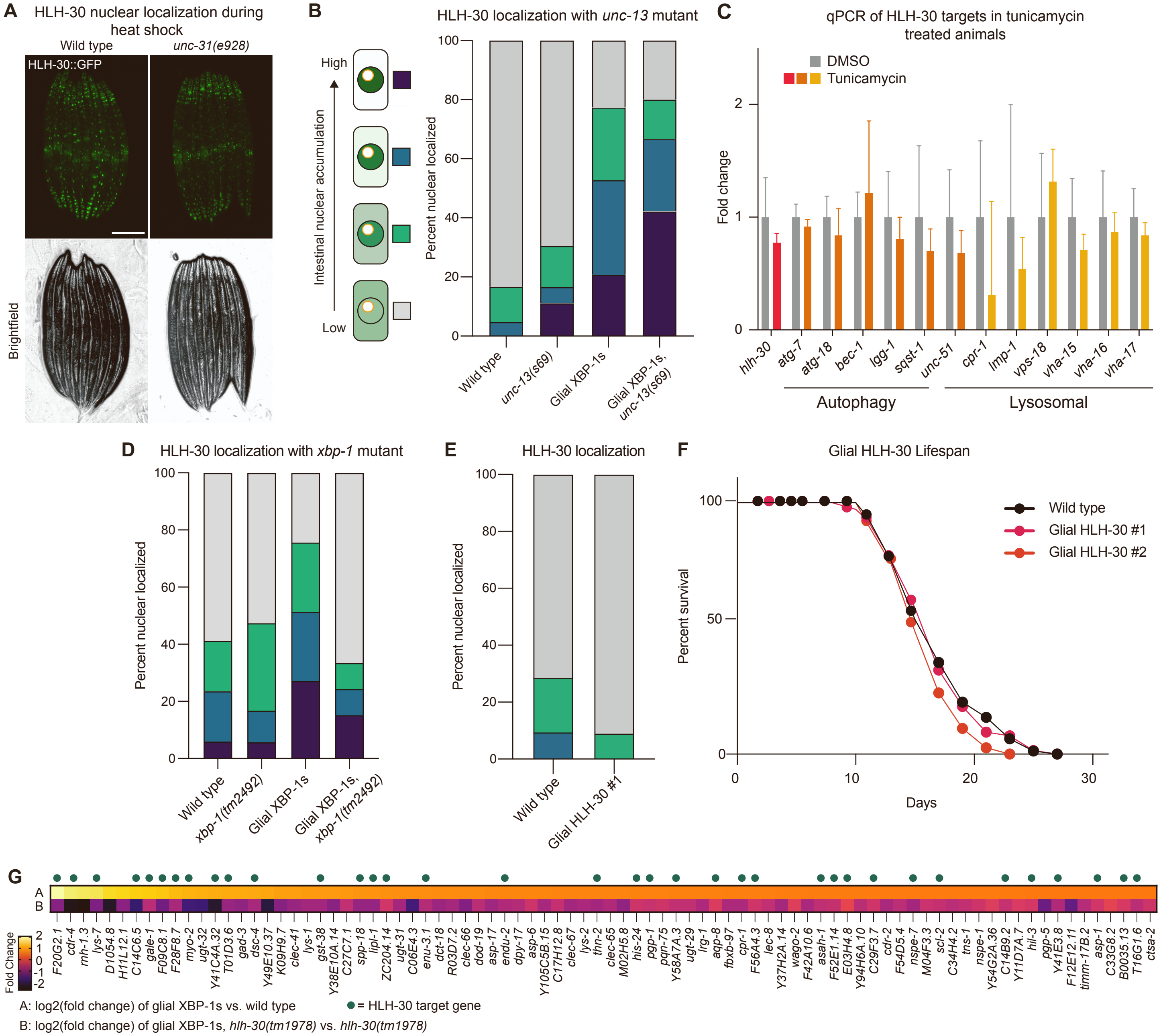
A
HLH-30 nuclear localization during heat shock
Wild type
unc-31(e928)
HLH-30::GFP
Brightfield
B
HLH-30 localization with unc-13 mutant
High
Low
Intestinal nuclear accumulation
Percent nuclear localized
0
20
40
60
80
100
Wild type
unc-13(s69)
Glial XBP-1s
Glial XBP-1s, unc-13(s69)
C
qPCR of HLH-30 targets in tunicamycin treated animals
DMSO
Tunicamycin
Fold change
0
1
2
hlh-30
atg-7
atg-18
bec-1
lgg-1
sqst-1
unc-51
cpr-1
lmp-1
vps-18
vha-15
vha-16
vha-17
Autophagy
Lysosomal
D
HLH-30 localization with xbp-1 mutant
Percent nuclear localized
Wild type
xbp-1(tm2492)
Glial XBP-1s
Glial XBP-1s, xbp-1(tm2492)
E
HLH-30 localization
Percent nuclear localized
Wild type
Glial HLH-30 #1
F
Glial HLH-30 Lifespan
Percent survival
0
50
100
Days
0
10
20
30
Wild type
Glial HLH-30 #1
Glial HLH-30 #2
G
A
B
Fold Change
2
1
0
-1
-2
F20G2.1
cdr-4
rnh-1.3
lys-7
D1054.8
H11L12.1
C14C6.5
gale-1
F09C8.1
F28F8.7
myo-2
ugt-32
Y41C4A.32
T01D3.6
gad-3
dsc-4
Y49E10.37
K09H9.7
clec-41
lys-1
gst-38
Y38E10A.14
C27C7.1
spp-18
lipl-1
ZC204.14
ugt-31
C06E4.3
enu-3.1
dct-18
R03D7.2
clec-66
dod-19
asp-17
endu-2
dpy-17
asp-6
Y105C5B.15
C17H12.8
clec-67
lys-2
thn-2
clec-65
M02H5.8
his-24
pgp-1
pqn-75
Y58A7A.3
ugt-29
lrg-1
aqp-8
fbxb-97
cpr-1
F56A4.3
lec-8
Y37H2A.14
wago-2
F42A10.6
asah-1
F52E1.14
E03H4.8
Y94H6A.10
C29F3.7
cdr-2
F54D5.4
nspe-7
M04F3.3
scl-2
C34H4.2
tns-1
nspe-1
Y54G2A.36
C14B9.2
Y11D7A.7
hil-3
pgp-5
Y41E3.8
F12E12.11
timm-17B.2
asp-1
C33G8.2
B0035.13
T16G1.6
ctsa-2
A: log2(fold change) of glial XBP-1s vs. wild type
= HLH-30 target gene
B: log2(fold change) of glial XBP-1s, hlh-30(tm1978) vs. hlh-30(tm1978)

Figure S4

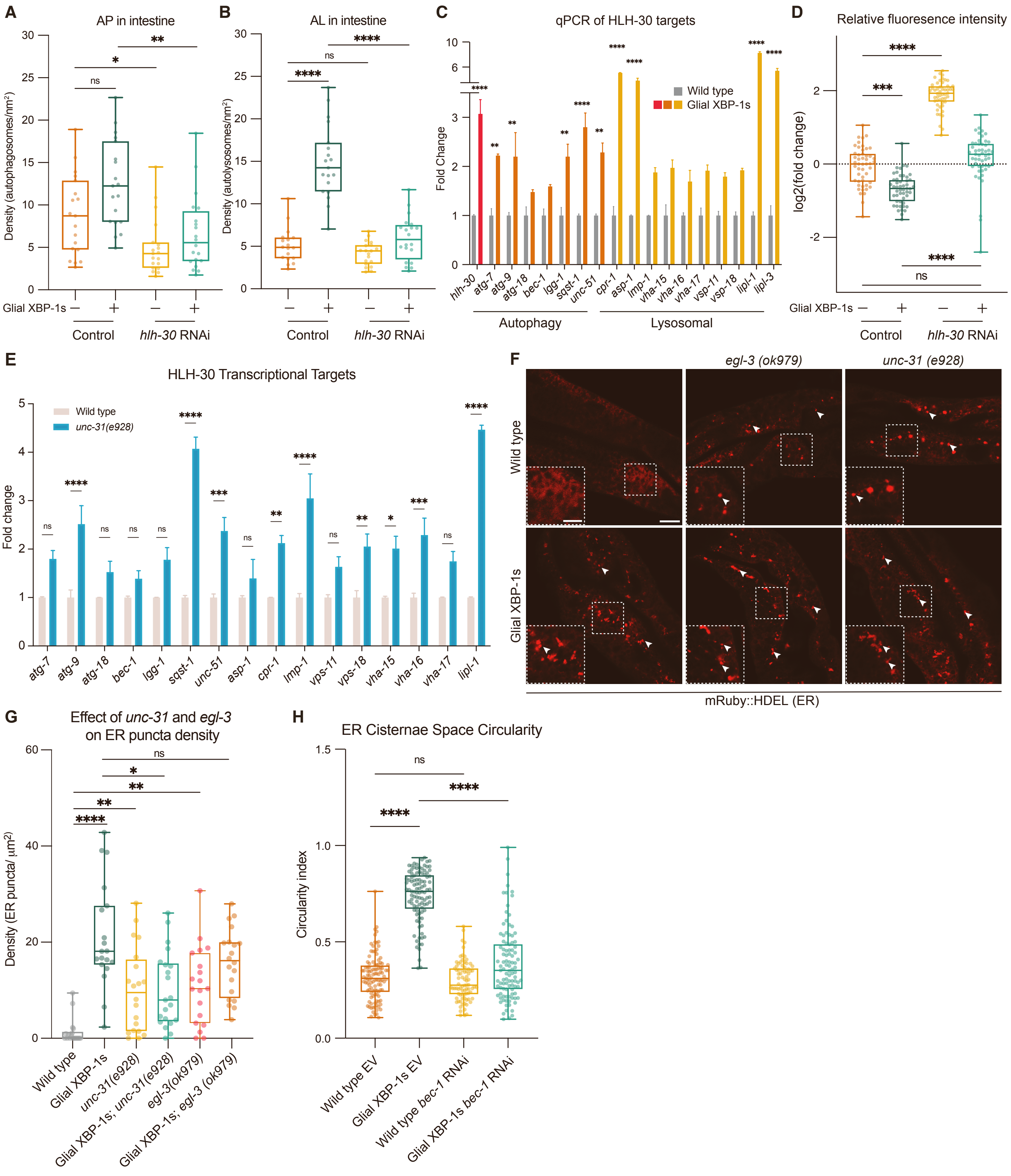

A
AP in intestine
Density (autophagosomes/nm²)
Glial XBP-1s
Control
hlh-30 RNAi
B
AL in intestine
Density (autolysosomes/nm²)
Control
hlh-30 RNAi
C
qPCR of HLH-30 targets
Fold Change
Wild type
Glial XBP-1s
Autophagy
Lysosomal
hlh-30
atg-7
atg-9
atg-18
bec-1
lgg-1
sqst-1
unc-51
cpr-1
asp-1
lmp-1
vha-15
vha-16
vha-17
vsp-11
vsp-18
lipl-1
lipl-3
D
Relative fluorescence intensity
log2(fold change)
Glial XBP-1s
Control
hlh-30 RNAi
E
HLH-30 Transcriptional Targets
Fold change
Wild type
unc-31(e928)
atg-7
atg-9
atg-18
bec-1
lgg-1
sqst-1
unc-51
asp-1
cpr-1
lmp-1
vps-11
vps-18
vha-15
vha-16
vha-17
lipl-1
F
egl-3 (ok979)
unc-31 (e928)
Wild type
Glial XBP-1s
mRuby::HDEL (ER)
G
Effect of unc-31 and egl-3 on ER puncta density
Density (ER puncta/ µm²)
Wild type
Glial XBP-1s
unc-31(e928)
Glial XBP-1s; unc-31(e928)
egl-3(ok979)
Glial XBP-1s; egl-3 (ok979)
H
ER Cisternae Space Circularity
Circularity index
Wild type EV
Glial XBP-1s EV
Wild type bec-1 RNAi
Glial XBP-1s bec-1 RNAi

SUPPLEMENTAL TABLES

Figure	Number for statistical analysis	Strain	Strain number	Median lifespan (days)	#death/ total	p-values, log-rank (Mantel-Cox)	p-values, log-rank (Mantel-Cox)	p-values, log-rank (Mantel-Cox)
						Compared to N2 (1)	Compared to glial XBP-1s (2)	Compared to RNAi/ mutant control (3)
2A	1	N2, empty vector RNAi	N2	15	80/100	n/a	<0.0001	<0.0001
2A	2	*hlh-17p::xbp-1s*, line 2, EV RNAi	AGD1724	21	65/100	<0.0001	n/a	<0.0001
2A	3	*N2, daf-16* RNAi	N2	12	79/100	<0.0001	<0.0001	n/a
2A	4	*hlh-17p::xbp-1s*, line 2, *daf-16* RNAi	AGD1724	19	62/100	<0.0001	<0.0001	<0.0001
2B	1	N2, empty vector RNAi	N2	17	100/120	n/a	<0.0001	<0.0001
2B	2	*hlh-17p::xbp-1s*, line 2, EV RNAi	AGD1724	22	80/120	<0.0001	n/a	<0.0001
2B	3	N2, *pha-4* RNAi	N2	13	98/120	<0.0001	<0.0001	n/a
2B	4	*hlh-17p::xbp-1s*, line 2, *pha-4* RNAi	AGD1724	17	88/120	0.9399	<0.0001	<0.0001
2C	1	N2, empty vector RNAi	N2	17	100/120	n/a	<0.0001	0.7165
2C	2	*hlh-17p::xbp-1s*, line 2, EV RNAi	AGD1724	22	80/120	<0.0001	n/a	<0.0001
2C	3	N2, *aak-1* RNAi	N2	20	103/120	0.7165	<0.0001	n/a
2C	4	*hlh-17p::xbp-1s*, line 2, *aak-1* RNAi	AGD1724	22	91/120	0.0003	0.2411	<0.0001
2D	1	N2, empty vector RNAi	N2	17	100/120	n/a	<0.0001	0.3701
2D	2	*hlh-17p::xbp-1s*, line 2, EV RNAi	AGD1724	22	80/120	<0.0001	n/a	0.0002
2D	3	*N2, aak-2* RNAi	N2	18	104/120	0.3701	0.0002	n/a
2D	4	*hlh-17p::xbp-1s*, line 2, *aak-2* RNAi	AGD1724	22	87/120	<0.0001	0.8428	<0.0001
2E	1	N2, empty vector RNAi	N2	16.5	106/120	n/a	<0.0001	0.2041
2E	2	*hlh-17p::xbp-1s*, line 1, EV RNAi	AGD1723	22	92/120	<0.0001	n/a	<0.0001
2E	3	N2, *hlh-30* RNAi	N2	17	113/120	0.2041	<0.0001	n/a
2E	4	*hlh-17p::xbp-1s*, line 1, *hlh-30* RNAi	AGD1723	18	84/120	0.0008	0.0659	<0.0001
2F	1	N2	N2	18	104/120	n/a	<0.0001	<0.0001
2F	2	*hlh-17p::xbp-1s*, line 2	AGD1724	24	84/120	<0.0001	n/a	<0.0001
2F	3	*hlh-30(tm1978)*	AGD2348	14	104/120	<0.0001	<0.0001	n/a
2F	4	*hlh-17p::xbp-1s*, line 2, *hlh-30(tm1978)*	AGD2491	16	98/120	<0.0001	<0.0001	<0.0001
S3F	n/a	N2	N2	17	83/120	n/a	n/a	n/a
S3F	n/a	*hlh-17p::hlh-30*, line 1 (array)	AGD2669	17	69/120	0.8665	n/a	n/a
S3F	n/a	*hlh-17p::hlh-30*, line 2 (array)	AGD2671	15	79/120	0.029	n/a	n/a
4D	1	N2, empty vector RNAi	N2	20	91/210	n/a	<0.0001	0.2315
4D	2	*hlh-17p::xbp-1s*, line 2, EV RNAi	AGD1724	24	82/120	<0.0001	n/a	<0.0001
4D	3	*N2, bec-1* RNAi	N2	20	100/120	0.2315	<0.0001	n/a
4D	4	*hlh-17p::xbp-1s*, line 2, *bec-1* RNAi	AGD1724	20	80/120	0.8619	<0.0001	0.2667
4E	1	N2, empty vector RNAi	N2	18	103/120	n/a	<0.0001	<0.0001
4E	2	*hlh-17p::xbp-1s*, line 2, EV RNAi	AGD1724	22	83/120	<0.0001	n/a	<0.0001
4E	3	N2, *atg-18* RNAi	N2	14	86/120	<0.0001	<0.0001	n/a
4E	4	*hlh-17p::xbp-1s*, line 2, *atg-18* RNAi	AGD1724	14	67/120	<0.0001	<0.0001	0.4091

Table S1. Lifespan statistics, related to Figures 2, S3, and 4. Summary of data from representative lifespan experiments displayed in figures.
